# Uniqueness of local myocardial strain patterns with respect to activation time and contractility of the failing heart: a computational study

**DOI:** 10.1186/s12938-018-0614-1

**Published:** 2018-12-05

**Authors:** Borut Kirn, John Walmsley, Joost Lumens

**Affiliations:** 1 Department of Physiology, Medical Faculty, University of Ljubljana, Zaloska 4, 1000 Ljubljana, Slovenia; 2 Department of Biomedical Engineering, CARIM School for Cardiovascular Diseases, Maastricht University Medical Center, Maastricht, The Netherlands

**Keywords:** Activation time, Cardiac strain, Computer simulation, Contractility, Reverse engineering, System degeneration

## Abstract

**Background:**

Myocardial deformation measured by strain is used to detect electro-mechanical abnormalities in cardiac tissue. Estimation of myocardial properties from regional strain patterns when multiple pathologies are present is therefore a promising application of computer modelling. However, if different tissue properties lead to indistinguishable strain patterns (‘degeneracy’), the applicability of any such method will be limited. We investigated whether estimation of local activation time (AT) and contractility from myocardial strain patterns is theoretically possible.

**Methods:**

For four different global cardiac pathologies local myocardial strain patterns for 1025 combinations of AT and contractility were simulated with a computational model (CircAdapt). For each strain pattern, a cohort of similar patterns was found within estimated measurement error using the sum of least-squared differences. Cohort members came from (1) the same pathology only, and (2) all four pathologies. Uncertainty was calculated as accuracy and precision of cohort members in parameter space. Connectedness within the cohorts was also studied.

**Results:**

We found that cohorts drawn from one pathology had parameters with adjacent values although their distribution was neither constant nor symmetrical. In comparison cohorts drawn from four pathologies had disconnected components with drastically different parameter values and accuracy and precision values up to three times higher.

**Conclusions:**

Global pathology must be known when extracting AT and contractility from strain patterns, otherwise degeneracy occurs causing unacceptable uncertainty in derived parameters.

**Electronic supplementary material:**

The online version of this article (10.1186/s12938-018-0614-1) contains supplementary material, which is available to authorized users.

## Background

In the healthy heart, deformation of the ventricular myocardium is relatively uniform. In contrast, many heart failure patients exhibit non-uniform myocardial deformation that may arise from heterogeneities in tissue properties such as activation time (AT) and contractility [1–3]. Non-invasively recorded myocardial deformation patterns may therefore be considered a source of important diagnostic information [4]. Determination of regional myocardial AT and contractility is of particular clinical relevance for heart failure patients with an indication for cardiac resynchronization therapy (CRT). Clinical studies have shown that a patients response to CRT depends on both the amount and type of electrical activation delay at baseline [5], and on the amount and location of scarred tissue present [6, 7]. Furthermore, positioning of the left ventricular (LV) lead in or near scarred myocardium is associated with reduced benefit from CRT [8].

Currently, peak strain, peak strain rate, and onset time of shortening are used in the clinic to measure regional contractility and activation time from strain recordings. These parameters have limitations as they can be influenced by both activation time and regional changes in contractility [3]. Computational models of the heart can directly relate regional myocardial tissue properties to local myocardial deformation. It may therefore be possible to estimate local AT and contractility from clinical strain pattern by fitting a computational model to reproduce the observed myocardial deformation.

The most common use for fitting to date is patient-specific computer modeling [9–12]. Currently, the models are used for fitting contractility or AT but not for both together. Elaborate fitting techniques are used to reduce the number of required simulations because each simulation is time consuming. For fitting contractility a global approach is used based on LV ejection fraction or invasively measured LV cavity pressures and volumes [13–15]. Data assimilation approaches that fit regional tissue contractility using unscented Kalman filters [16, 17] or gradient-based minimization procedures [18, 19] through comparing simulated regional myocardial deformation with cine MRI have also been shown to be tractable, raising the possibility of extracting regional tissue properties via a model.

AT is not generally taken into account when fitting a model to regional mechanical deformation, and is normally determined based on invasive electrical mapping. Fitting of regional AT is known to carry a degree of uncertainty arising from measurement noise. Konukoglu et al. [20] and Wallman et al. [21] have addressed this uncertainty using Bayesian approaches for estimation of electrophysiological conductivity parameters from endocardial activation maps. Statistical learning has also been used to estimate myocardial diffusivities based on the 12-lead electrocardiogram in patients with dilated cardiomyopathy [22].

Cardiac wall mechanics are dependent on many tissue and geometry parameters with non-linear relationships. The result may be that radically different sets of parameters can lead to indistinguishably similar local myocardial deformation patterns, given a realistic level of measurement error. The uncertainty in any extracted tissue parameters therefore risks becoming impractically large when fitting to real data. This degeneracy in model behavior with respect to model parameters is well-known throughout systems biology [23, 24], and has also been demonstrated in a cardiac context in electrophysiological models [25, 26].

Our aim is to establish whether or not the estimation of local AT and contractility based on myocardial strain patterns is theoretically possible by investigating whether degeneracy in regional mechanical deformation patterns can occur when AT and contractility are varied. We map degeneracy in AT and contractility using parameter space exploration and simulations of regional mechanical deformation using Hill based computational model of cardiac mechanics and cardiovascular dynamics [27]. The model has previously been shown to provide simulations of myocardial strain in the failing heart with regional differences in AT and contractility [28, 29], and is a fast alternative for simulation of cardiac mechanics and cardiovascular system hemodynamics as compared to finite element-based cardiac models [27, 30, 31].

In this study, we determine parameter values that generate strain patterns similar to those arising from simulations using any other parameter values within the parameter space. Similarity is determined based on direct comparison within a realistic amount of measurement noise. Using connected component analysis, we determine whether these parameters lie in similar regions of parameter space as well as the size and shape of these regions. Our analysis is performed for four common global cardiac pathologies: global heart failure (HF) alone and with a severely hypocontractile region (HF + HYPO), a left bundle branch block activation pattern (HF + LBBB), and the combination thereof (HF + HYPO + LBBB).

## Methods

### General description of the computational model

**Fig. 1:**
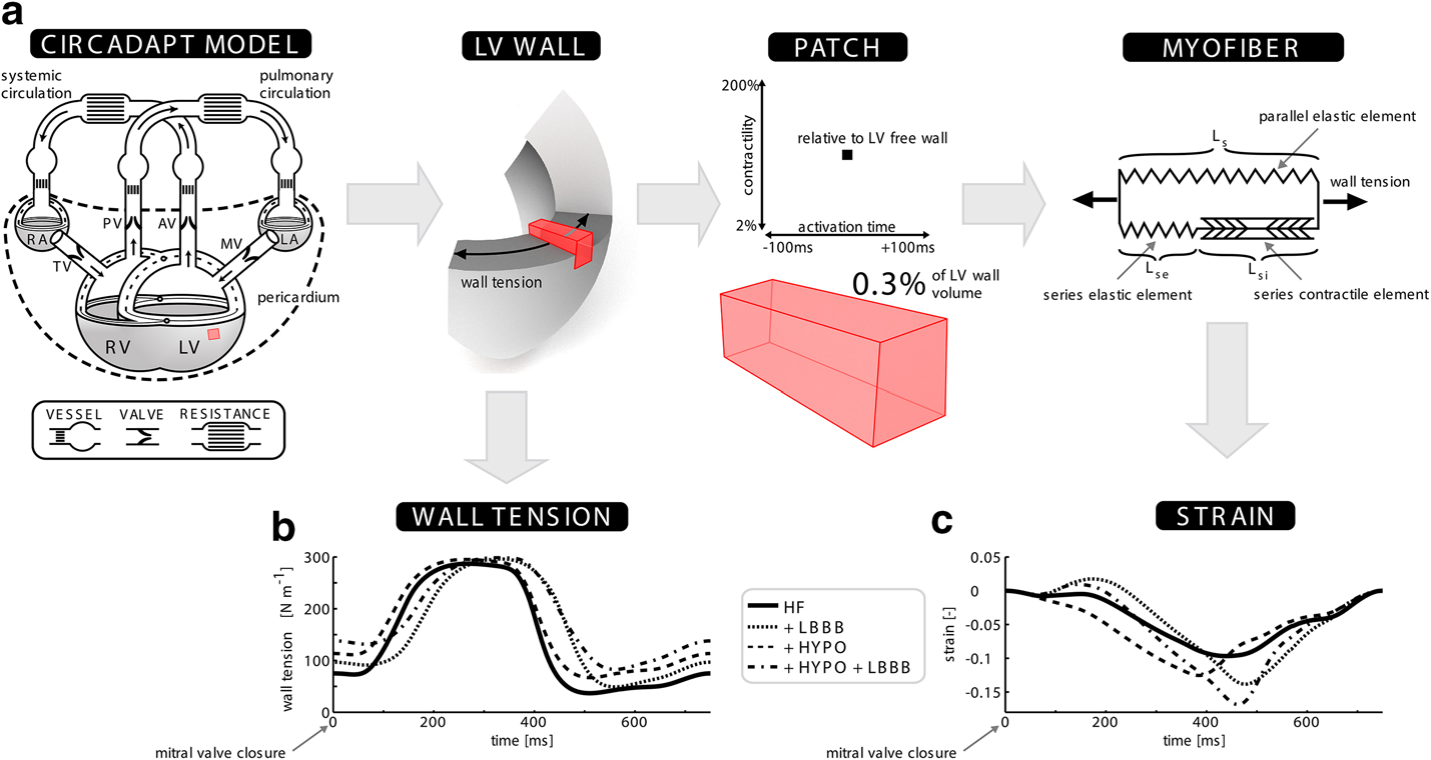
**a** A schematic representation of the model (adapted from Lumens et al. with permission). The relation between the scales that are relevant to this study is also shown (LV wall, small tissue patch, myofiber). Altered mechanical deformation in the small patch taking up 0.3% of the volume of the left ventricular (LV) free wall is simulated by changing the activation time by 100 ms relative to the majority of the wall, and the contractility between 2 and 200% of the value for the majority of the LV free wall. **b** The wall tension traces from the LV free wall for the heart failure (HF), hypocontractility (HF + HYPO.), left bundle-branch block (HF + LBBB), and left bundle-branch block with hypocontractility (HF + LBBB + HYPO) simulations. **c** Myofiber strain in the small patch from each simulation, with parameters in the patch equal to those in the majority of the LV free wall

Details on the underlying assumptions and concepts of the computational model (CircAdapt, http://www.circadapt.org) have been previously published elsewhere [25, 28]. Briefly, the model consists of different modules, including myocardial walls, cardiac valves, large blood vessels, systemic and pulmonary peripheral vasculature, and the pericardium. These modules are coupled to represent the closed-loop cardiovascular system (Fig. 1a). Mechanical interaction between the ventricles is incorporated through the equilibrium of tensile forces at the junction of the three myocardial walls i.e., the LV free wall, the interventricular septum, and the right ventricular (RV) free wall [30]. Active and passive myocardial tissue behavior is described using the sarcomere model outlined below. Global pump mechanics (in terms of cavity pressure and volume) of all four cardiac chambers are related to local myofiber mechanics (in terms of Cauchy myofiber stress and natural myofiber strain) in the myocardial walls by the principle of conservation of energy [27]. Tissue anisotropy can be neglected during the calculation of fiber stress and strain following the one fiber model of Arts et al. [32].

### Sarcomere model

The sarcomere model relates changes in AT and contractility to changes in sarcomere length, and hence strain. A modified three-element Hill model of myofiber contraction is used to calculate local fiber Cauchy stress and stiffness from natural fiber strain [30, 31] (Fig. 1a: right panel). Total sarcomere length (*L*_*s*_) is the sum of the intrinsic sarcomere length (*L*_*si*_) and the length of the series elastic element (*L*_*se*_). *L*_*si*_ represents the length the sarcomere would have if it were not under external load, and *L*_*se*_ represents the stretch of the sarcomere (in the cross bridges, titin, etc.) caused by an external load being applied. The contractility and the AT alter the behavior of the contractile element. The total active stress *σ*_*f,act,T*_ generated is given by,

1 $$\sigma_{{f,{\text{actT}}}} = \sigma_{{f,{\text{act}}}} C\left( {L_{si} - L_{{si,{\text{ref}}}} } \right)\frac{{L_{se} }}{{L_{{se,{\text{iso}}}} }} ,$$

where *σ*_*f,act*_ represents the capacity of the tissue to generate active stress. In this study, variations in contractility are achieved by changing *σ*_*f,act*_. *L*_*si,Ref*_ and *L*_*se,iso*_ are reference lengths. The variable *C* is a non-dimensional, phenomenological representation of the density of cross-bridge formation and its time-derivative is modeled as a function of *L*_*si*_ and time *t*, accounting for experimentally observed length dependence of activation and force–velocity relation of cardiac myofibers [33, 34].

2 $$\frac{dC}{dt} = f\left( {L_{si} ,t} \right)$$

Changing local AT alters the onset time of rise of *C* and, hence, determines local myocardial pre- and afterload. A full description of the sarcomere model has previously been published by Walmsley et al. [31].

### Relating wall tension to fiber strain

In the CircAdapt model, cavity volume and pressure are related to tissue deformation through wall tension and curvature as published by Lumens et al. [30]. Each myocardial wall may be broken up into patches. Within each patch, tissue parameters and mechanics are homogeneous, while parameters can differ between patches. The patches making up a wall are considered to act in series and so they share a common wall tension and curvature as described previously by Walmsley et al. [31]. Briefly, cavity volume allows calculation of wall area and curvature. The value of the intrinsic sarcomere length *L*_*si*_ is used to estimate stiffness in each patch and the unloaded area of each segment. Summing segmental unloaded areas and stiffness gives the wall unloaded area and wall stiffness. Wall area, wall stiffness, and wall unloaded area allow the calculation of the current wall tension that is common to all segments. The wall tension and curvature then allow calculation of transmural pressure through Laplaces law. Pressures determine flows and consequently the changes in cavity volumes. Furthermore, wall tension, segmental stiffness, and unloaded segment area allow calculation of the current area of each segment, and therefore the current local strain and stress in each segment. These properties are used to update the intrinsic sarcomere length in each segment.

### Baseline heart failure simulations

A simulation of normal cardiovascular mechanics and hemodynamics representing the healthy adult cardiovascular system under baseline resting conditions was obtained as described previously [29] and served as starting point for four different pathology simulations.

Firstly, a global heart failure (HF) simulation was obtained by decreasing myocardial contractility (f,act) of the three ventricular walls to 50% of its normal value, so that LV ejection fraction was 30%. All three ventricular walls were activated simultaneously, 200 ms after the right atrium. The resulting HF simulation was then used as starting point for the remaining three patient simulations. Secondly, a failing heart with left bundle-branch block (HF + LBBB) was simulated by delaying onset time of septal and LV free wall activation by 25 ms and 75 ms, respectively, relative to the RV free wall. Thirdly, a failing heart with regional LV hypocontractility (HF + HYPO) was simulated by subdividing the LV free wall in two equally sized patches [31] and decreasing contractility of one patch to 20% of its normal value. Finally, a failing heart with both LBBB and hypocontractility (HF + HYPO + LBBB) was simulated by combining both pathologies as described above.

In all simulations, systemic peripheral resistance and total blood volume were adjusted to represent physiological homeostatic control, so that mean arterial pressure and cardiac output were maintained at their resting values (92 mmHg and 4.2 L/min, respectively). Heart rate was kept constant at 80 beats/min.

### Local myofiber strain

To evaluate the effects of local AT and contractility on myocardial deformation, a small myocardial tissue segment, representing 0.3% of total LV wall mass, was created (Fig. 1a). The size of this segment was chosen so that changes of its tissue properties did not significantly affect global cardiac pump mechanics and hemodynamics. Therefore, the small segments mechanical boundary conditions, represented by the time course of wall tension, remained similar for all simulations within each of the four different baseline HF simulations introduced in the previous section.

The myofiber strain *E*_*f*_ in the small patch was calculated directly from the sarcomere length *L*_*s*_ using

3 $$E_{f} (t) = \frac{{L_{s} (t)}}{{L_{s,0} }} - 1$$

where *L*_*s,*0_ denotes the sarcomere length at the time of closure of the mitral valve. Sarcomere length was calculated using a 2 ms temporal resolution. Since tissue mechanics within a patch are homogeneous, *E*_*f*_ represents the average myocardial strain from this region of tissue.

### Parameter space exploration

For each baseline HF simulation, we performed 1025 different simulations, each with a different combination of AT and contractility in the small segment. AT was varied from − 100 to + 100 ms in 41 steps of 5 ms. The value of AT is given relatively to the time of activation of the large segment which means that when AT is negative the activation of small segment precedes the large segment and when AT is positive the activation of the small segment is delayed with respect to the large segment. In some patients with heart failure, for example those with LBBB, different regions of cardiac tissue may begin to contract considerably earlier or later than others. The contractility was scaled from 2 to 200% of the large segments value, in 25 equidistant steps of 8.25%.

### Strain pattern cohort

**Fig. 2:**
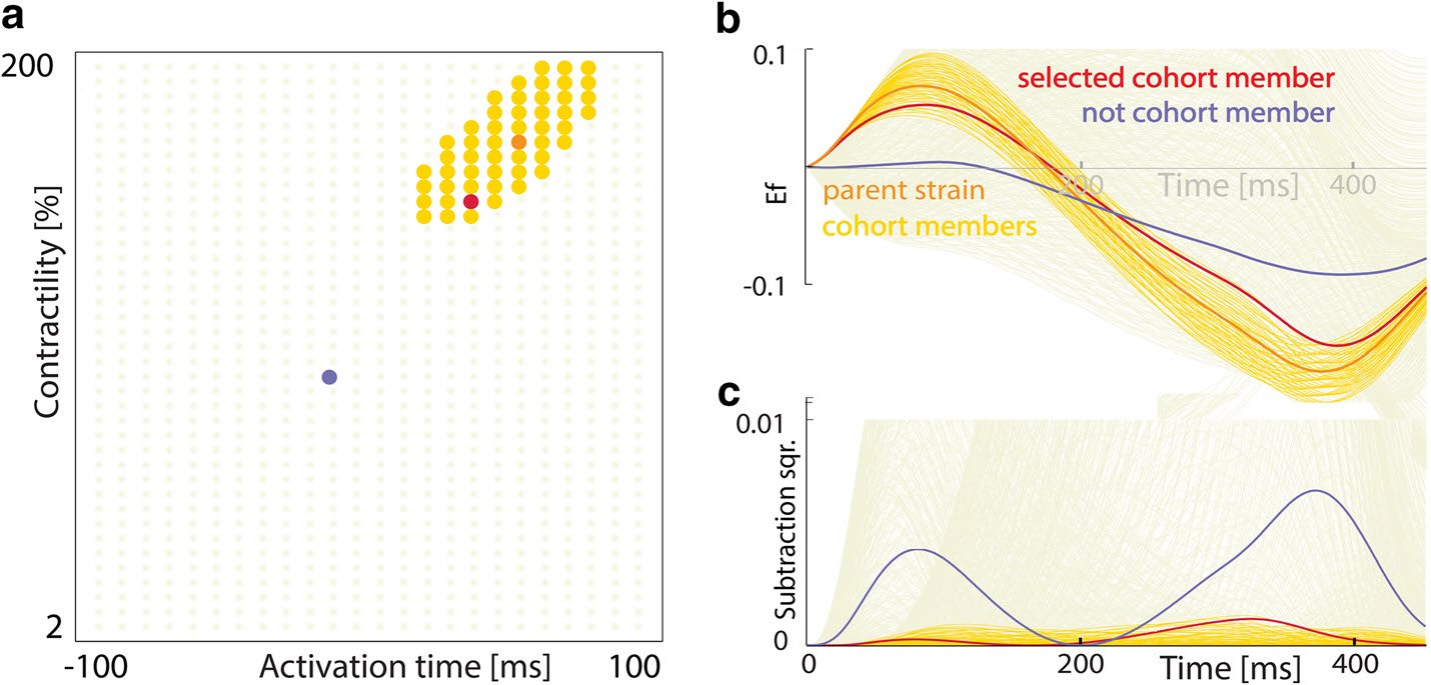
A graphical explanation of the definition of a cohort (yellow) for a given parent strain pattern (orange). **a** The cohort’s location in parameter space. The red and blue points highlight an example cohort member and non-member, respectively. **b** The strain patterns arising from the parameters highlighted in **a**. The time axis goes from mitral valve closure to mitral valve opening. **c** The corresponding squared-difference between the parent strain pattern and all other strain patterns at each time point

A realistic level of strain measurement error was emulated by determining a cohort of similar strain patterns. The similarity (*D*) of each strain pattern (*i*) to the reference parent strain pattern (*p*) was calculated as

4 $$D_{p,i} = \sqrt {\frac{{\mathop \sum \nolimits_{t = 1}^{N} \left( {E_{f,p,t} - E_{f,i,t} } \right)^{2} }}{N}}$$

where the sum of the squared strain differences (*E*_*f,p*_− *E*_*f,i*_)^2^ is calculated from mitral valve closure (*t *= 1) until mitral valve opening (*t *= *N*). The process was repeated using every simulated strain pattern as the parent strain pattern. A cohort was defined by a parent strain pattern p with all strain patterns i that satisfy *D*_*i,p*_≤ 0.02. The basis of this value is discussed in “Discussion” section. B. For the HF pathology simulations, uncertainty analysis was performed using two additional similarity thresholds (*D *≤ 0.01 and *D *≤ 0.03) to assess the influence of threshold level on cohort size. A description of the generation of a cohort is provided in Fig. 2.

Two different types of cohorts were assembled. In the first case, only strain patterns generated from the same baseline pathology situation (HF, LBBB, HYPO, or LBBB + HYPO) as the parent strain pattern were included in the cohort. In the second case, strain patterns generated from the other baseline pathologies were also included in the cohort. The first case reflects the situation when the global cardiac pathology is known and the second when the global cardiac pathology is unknown.

### Evaluation of uncertainty and degeneracy

The uncertainty of strain patterns in the parameter space was evaluated from the distance between the cohort members and parent strain pattern. Greater distance represents a larger uncertainty in how well a given strain pattern uniquely represents one set of parameters. The uncertainty was evaluated using both accuracy and precision, which were calculated for each parent strain pattern for AT and contractility, separately. The accuracy was calculated as the absolute difference between the parameter value of the parent strain and the average parameter value of the corresponding cohort members The precision was calculated as the standard deviation of the parameter values within the cohort.

We used connected component analysis (Matlab, The MathWorks Inc., Natick, Massachusetts, USA) to determine whether all parameters in the cohort were adjacent, meaning that all cohort members differed by not more than one parameter step from at least one another member from the cohort, i.e. ± 5 ms AT and 8% contractility, respectively.

## Results

**Fig. 3:**
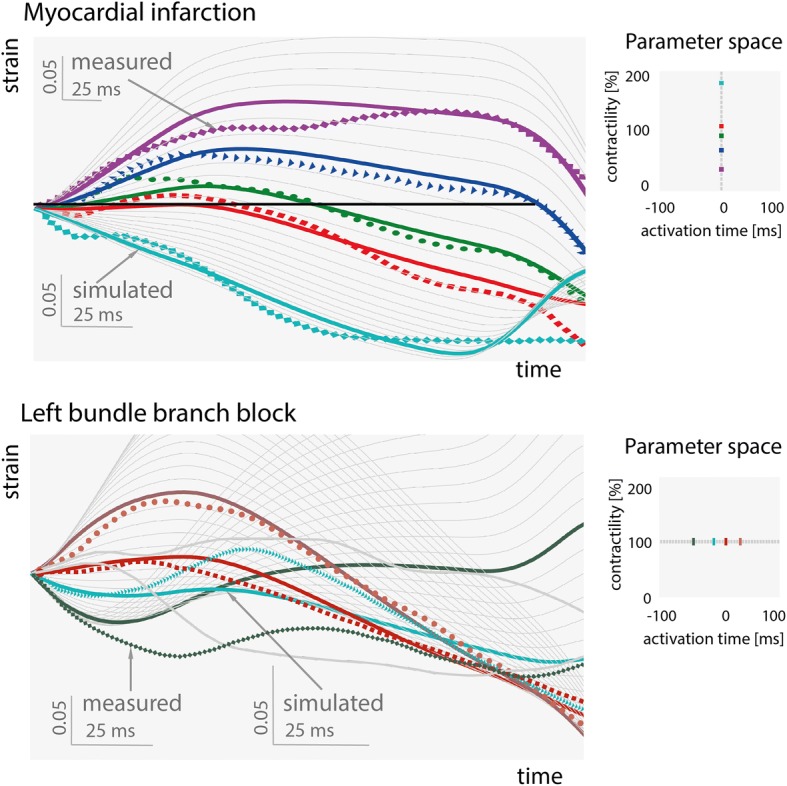
Comparison of simulated strain patterns (solid lines) with measured strain patterns (dotted lines) from patients with myocardial infarction (MI; upper panel) or left bundle branch block (LBBB; lower panel). Colored solid lines indicate the best match simulations. Note that the time and strain axes are scaled differently for measurements and simulations to facilitate qualitative comparison of strain patterns

Figure 3 demonstrates that simulations of local changes of contractility or onset time of activation could produce strain patterns similar to those clinically measured in patients with myocardial infarction (MI) and left bundle branch block (LBBB), respectively. The LBBB and MI data were originally published by Risum et al. [2] and Smiseth et al. [4], respectively. They measured longitudinal strains with speckle-tracking echocardiography (4-chamber view). Measurements from the patient with MI are compared with simulations of various levels of contractility and zero activation delay, while the LBBB measurements are compared with simulations of various activation times and normal contractility. In MI patient, for all five and in LBBB patient for four out of six measured strain patterns a matching simulation was found.

### Uncertainty within one global cardiac pathology

**Fig. 4:**
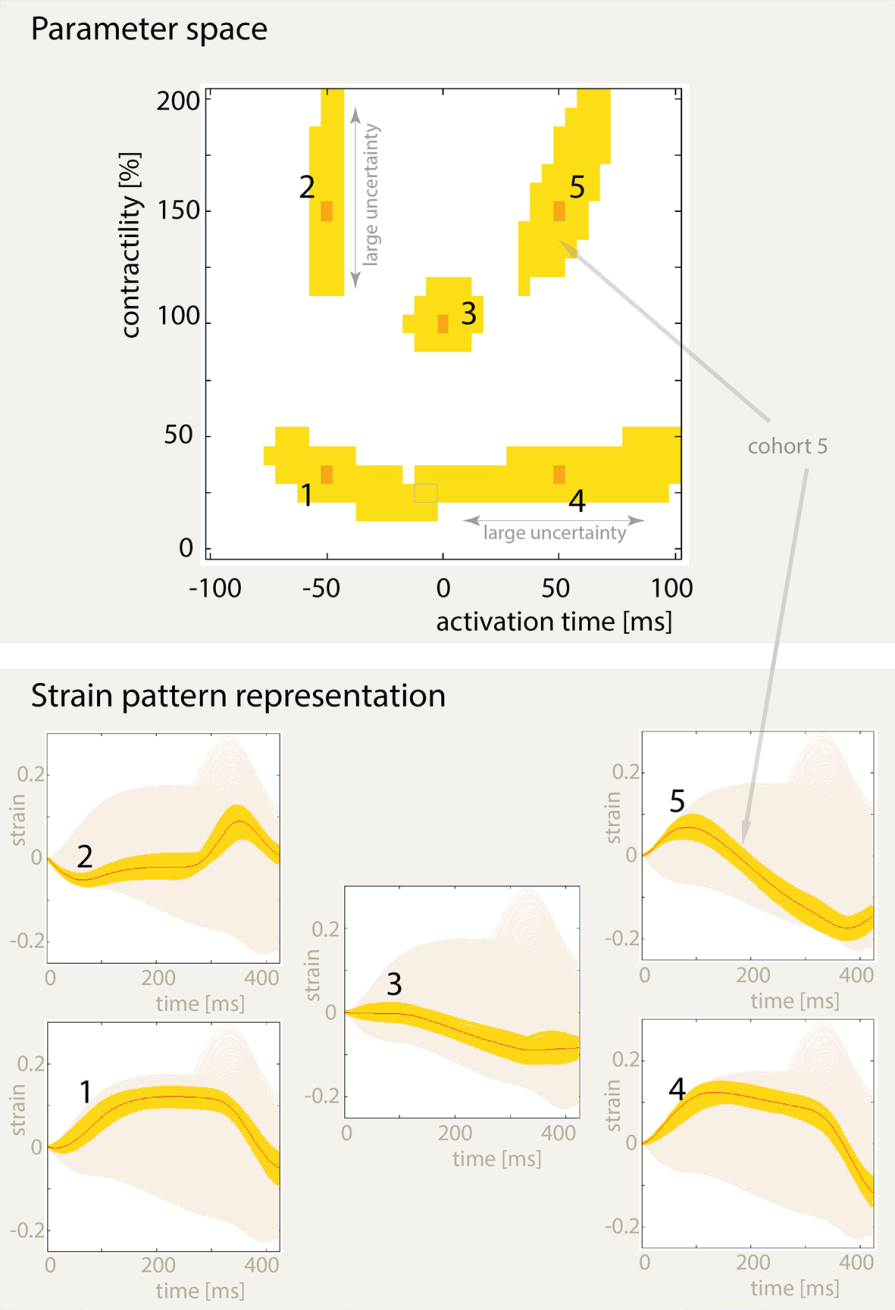
Parameter space (top) and strain pattern (bottom) representations of cohorts (yellow) generated by five (1–5) selected parent strain patterns (orange) for the global HF pathology. The cohorts were generated using a threshold of 0.02. Cohorts 1 and 4 have a small overlap region in the parameter space. Each cohort has a different shape and size. In the strain pattern representation, all 1025 simulated strain patterns from the HF pathology are shown as a light coloured background. The shapes of all cohorts in the parameter space are shown for all global pathologies in Additional files 1, 2, 3, 4

For the HF condition, all 1025 simulated strain patterns from the small segment are shown as shaded background signals in Fig. 4 (Strain pattern representation). The top panel of Fig. 4 shows five representative points in the parameter space with their cohort parameter component in yellow. The corresponding strain patterns are plotted in yellow in the bottom panel of Fig. 4.

Connected component analysis showed that the parameters in all cohorts form one connected component (island) in the parameter space (Fig. 4: top panel). The cohorts were not symmetrically distributed around the parent parameter set, and the shape of each cohort was different. The shape of cohorts 1 and 4 (low contractility) in the parameter space was elongated along the AT axis relative to the contractility axis, which indicates higher uncertainty in AT as compared to contractility. In contrast, the shape of cohorts 2 and 5 (high contractility) was elongated along the contractility axis relative to the AT axis, which indicates higher uncertainty in contractility. The cohort number 3, which is centered on the baseline simulation with standard AT and contractility has circular shape, thus uncertainty of AT and contractility is similar near the baseline parameters (AT = 0 ms, Contractility = 100%). Cohorts from different regions of the parameter space generally have distinctively different shapes. Consequently, the accuracy and precision were different for each location in the parameter space. Among baseline HF simulations (HF, HF + HYPO, HF + LBBB and HF + LBBB + HYPO) the shapes of cohort parameter components were similar. The shapes of all cohort components in the parameter space and the corresponding strain patterns are shown for all four baseline simulations in Additional files 1, 2, 3, 4.

Mean size of the cohorts depends on the measurement error. By tripling the measurement error from ≤ 0.01 to ≤ 0.03 the cohort size increased roughly 6 folds from 13 to 83 members. For baseline simulations, mean cohort size at measurement error ≤ 0.02 was 44, mean uncertainty of AT was 8.3 ms ± 17 ms (accuracy ± precision) and mean uncertainty of contractility was 6% ± 14%. The mean values for each individual baseline simulation are shown in Table 1.

**Table 1:** Mean uncertainty in parameter space within one global cardiac pathology (C: contractility, *absolute value)

Parent strain	Meas.	Cohort	Accuracy	Accuracy	Precision	Precision
Pathology	Error	Size	AT* (ms)	C* (%)	AT (ms)	C (%)
HF	± 0.01	13.1	4.6	2.3	10.6	7.7
	± 0.02	43.4	8.2	5.2	16.9	13.8
	± 0.03	83.1	11.6	8.3	22.0	18.9
HF + HYPO	± 0.02	46.3	9.2	5.3	18.2	13.3
HF + LBBB	± 0.02	41.0	7.2	5.9	16.1	14.0
HF + LBBB + HYPO	± 0.02	45.0	8.7	5.8	17.9	13.8

**Fig. 5:**
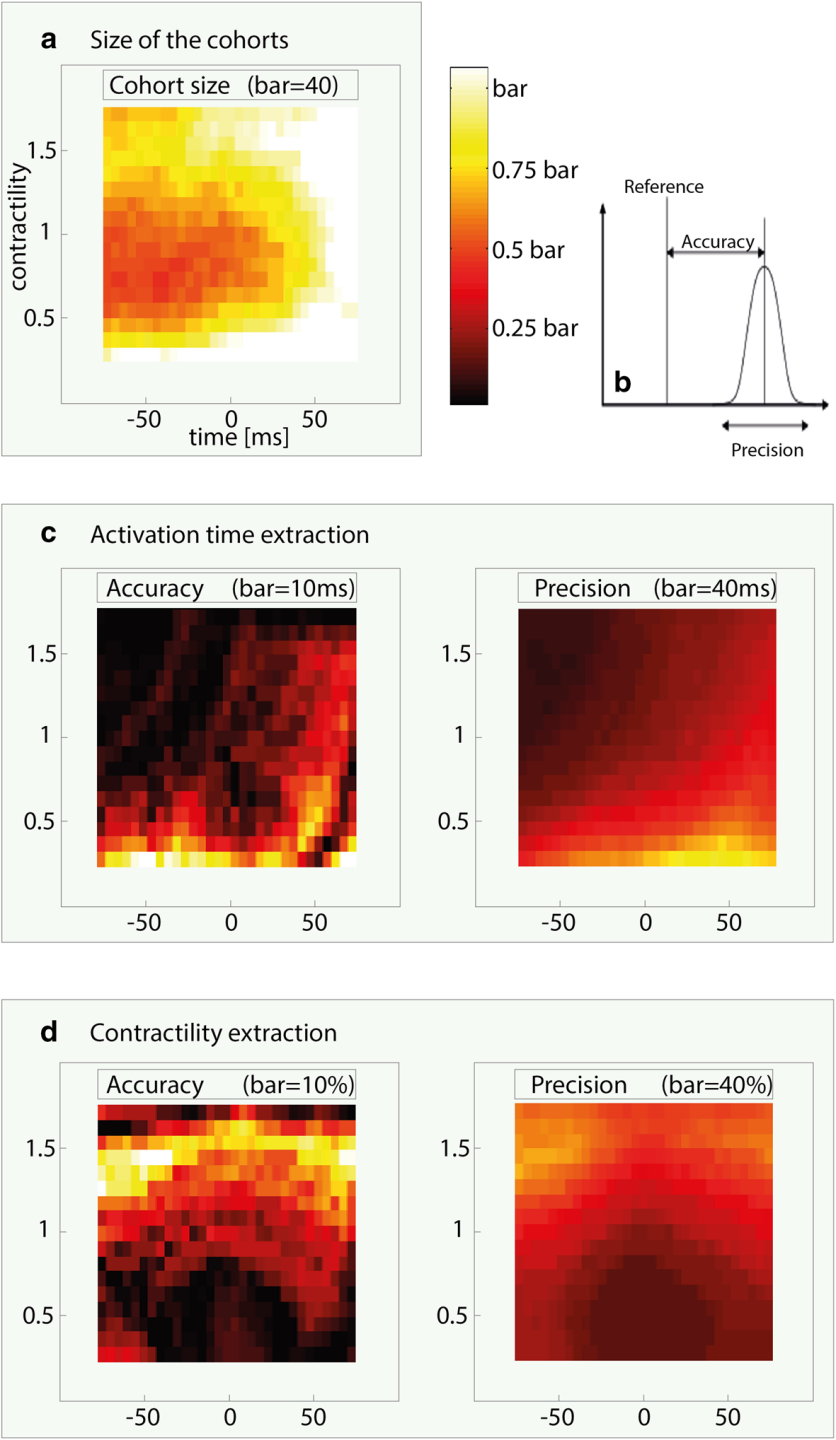
**a** The total size of the cohorts generated from each strain pattern in parameter space. Brighter colors indicate larger cohorts. The parameter scale determines the value of the colour bar in each plot (see title of each plot). **b** A diagrammatic representation of accuracy and precision. **c**, **d** Uncertainty in AT and contractility, respectively, as evaluated by accuracy and precision. Brighter colors indicate lower accuracy or precision. The parameter space shown in all plots was narrowed to fit the range from − 75 to 75 ms and 25% to 175% for AT and contractility, respectively, to prevent border effects from affecting the results

The distribution of the uncertainty and the cohort size across the parameter space is shown in Fig. 5 for the global HF simulation. These distributions were similar for the HF + LBBB, HF + HYPO, and HF + LBBB + HYPO cases. In general, the following basic characteristics were observed: the cohorts (Fig. 5a) tended to contain more members when the AT was >+ 50 ms, or when contractility was low; higher accuracy and precision of AT were associated with higher contractility values and lower AT, as indicated by the darker region in the upper-left hand corners in the accuracy and precision plots in Fig. 5c; higher accuracy and precision of contractility were associated with low contractility when AT was > − 50 ms or < + 50 ms, as illustrated by the dark regions in the middle of the lower halves of the accuracy and precision plots in Fig. 5d.

### Uncertainty among all four global cardiac pathologies

When including all strain patterns from all four pathologies simulated, the cohorts were larger and uncertainty was up to three times greater (Table 2) as compared to when only one baseline HF simulation was considered (Table 1). By tripling the measurement error from ≤ 0.01 to ≤ 0.03 the cohort size increased ninefold from 16 to 152 members. For baseline simulations, mean cohort size at measurement error ≤ 0.02 was 83, mean uncertainty of AT was 18.6 ms ± 26.1 ms (accuracy ± precision) and mean uncertainty of contractility was 13.2% ± 20.2%.

**Table 2:** Mean uncertainty in parameter space within combined four global cardiac pathologies (C: contractility, *absolute value)

Parent strain	Meas.	Cohort	Accuracy	Accuracy	Precision	Precision
Pathology	Error	Size	AT* (ms)	C* (%)	AT (ms)	C (%)
HF	± 0.01	16.2	8.7	7.8	14.9	14.0
	± 0.02	76.6	16.4	14.9	21.7	21.1
	± 0.03	152.2	19.2	20.0	26.0	27.3
HF + HYPO	± 0.02	87.7	17.2	10.3	27.7	21.0
HF + LBBB	± 0.02	81.6	22.5	13.8	25.3	19.7
HF + LBBB + HYPO	± 0.02	86.4	18.3	14.0	29.5	19.1

**Fig. 6:**
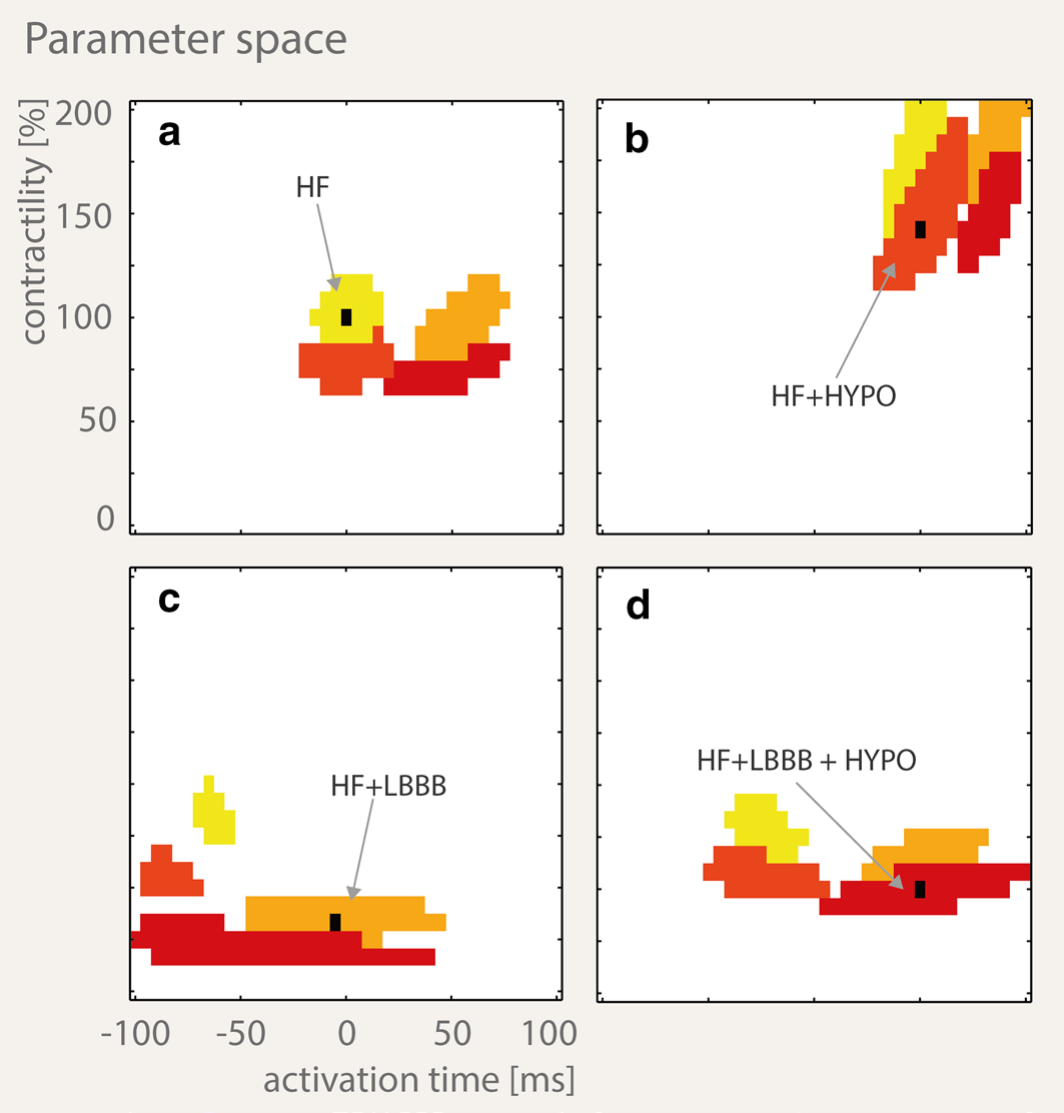
**a**–**d** Four cohorts obtained when allowing strain patterns from all four global cardiac pathologies to be include in the cohort. The parent strain pattern (black) is surrounded by a component with same pathology as the parent strain pattern. **a** HF (yellow), **b** HF + HYPO (orange/red), **c** HF + LBBB (orange), **d** HF + LBBB + HYPO (red). The cohorts also contain strain patterns arising from other pathologies, shown in their corresponding color. Some of the resulting components are not connected to the component generated by the original pathology. All 4100 parent strain patterns and their corresponding cohorts are shown in Additional files 1, 2, 3, 4

Figure 6 shows four examples of combined cohorts each with a parent strain pattern from one of the four HF simulations (Fig. 5a–d: black points). The parent strain pattern of the cohort in Fig. 6a is the same as the one for cohort 3 in Fig. 4, both belonging to the global HF simulation. While the HF component is the same in both figures, the cohort in Fig. 6a contains additional components composed of strain patterns obtained from all additional three pathologies. The example shown in Fig. 6c demonstrated degeneracy in the AT and contractility values that generated similar strain patterns when multiple pathologies were considered, using an error threshold of ≤ 0.02. The degeneracy can be appreciated from the non-connected components in Fig. 6c where two groups of simulations with drastically different parameter sets yield similar strain patterns. In Fig. 6c, a parent strain from the HF + LBBB simulation (orange) with AT = − 5 ms and contractility = 35% is within measurement error ≤ 0.02 similar to a dislocated group of HF simulations (yellow) with drastically different parameters: mean AT = − 65 ms and mean contractility 88%.

**Fig. 7:**
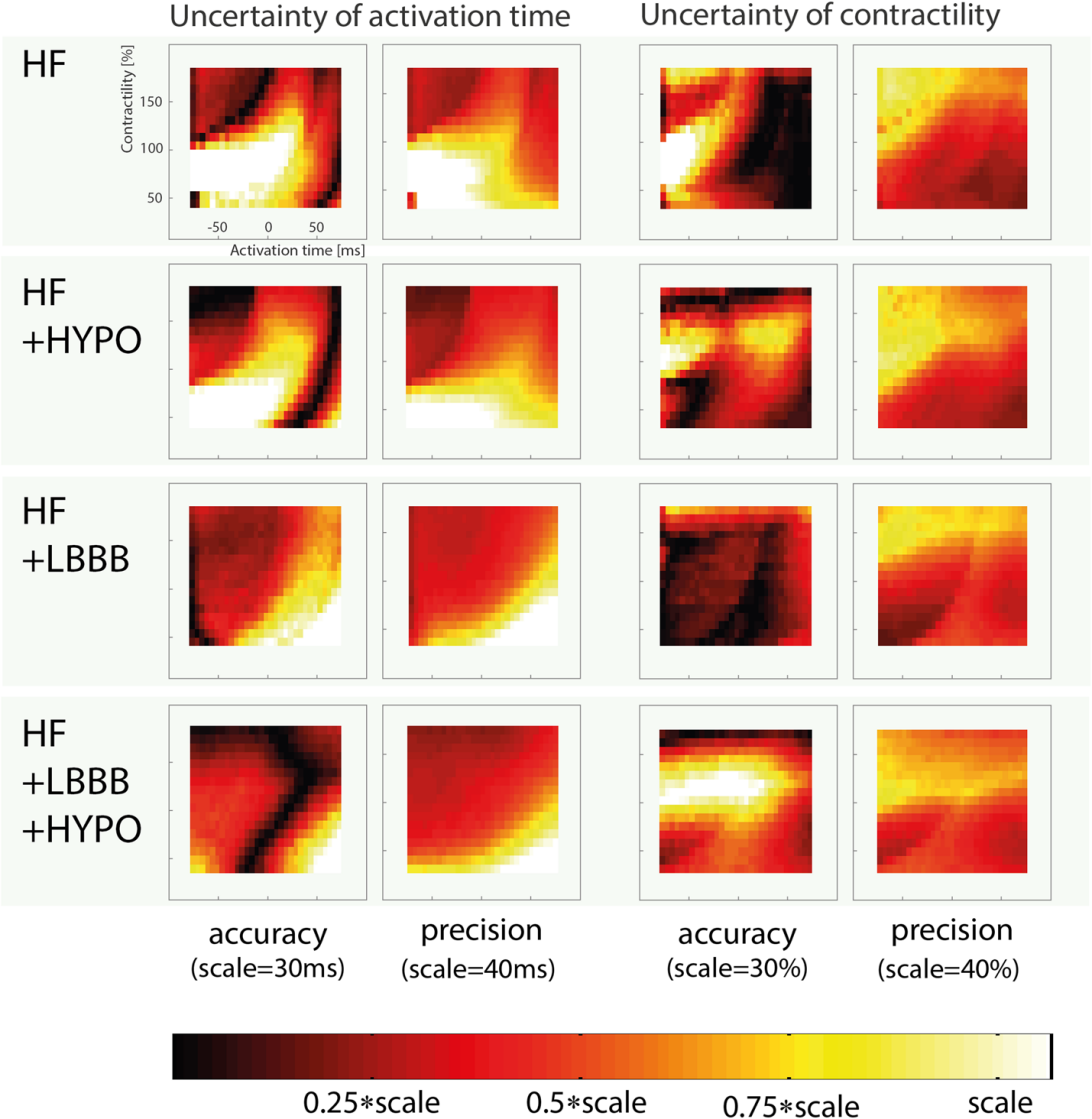
The distribution of uncertainty in AT and contractility when strain patterns from other global HF simulations are included in each cohort are shown for each of the four global HF simulations (HF, HF + HYPO, HF + LBBB, and HF + LBBB + HYPO). Brighter colors indicate lower accuracy or precision. The parameter scale determines the value of the color bar in each plot (see title of each plot). The parameter space shown in all plots was narrowed to − 75 ms to 75 ms and 25% to 175% for AT and contractility, respectively, to prevent border effects from affecting the results

The uncertainty distribution plots in Fig. 7 show that uncertainty as measured by accuracy and precision is distributed differently for each of the underlying pathologies. In all four pathologies, the uncertainty in AT was highest when contractility was low (< 100%). For the HF and HF + HYPO conditions, uncertainty in AT was highest when the tissue was early activated (AT < 0 ms), whereas for HF + LBBB and HF + LBBB + HYPO uncertainty was highest when the tissue is delayed in activation (AT > 0 ms). The uncertainty in contractility, in terms of accuracy, was different for each of the underlying pathologies. In contrast, precision of contractility was similarly distributed over the parameter space for all four pathologies, and tended to be lower where AT was earlier and contractility was higher.

## Discussion

In this computational study, we found that when the global cardiac pathology is known, local myocardial strain patterns uniquely represent realistic values of activation time and contractility. Thus no theoretical limitation was found for extracting local tissue properties from local strain measurements. When the global pathology is not known, however, the system becomes degenerated and strain patterns can be similar despite large differences in the tissues AT and contractility. Consequently the uncertainty of AT and contractility becomes large which makes extraction of tissue parameters impractical.

Our study confirms that any methodology used for extraction of mechanical and electrical myocardial tissue properties from local deformation patterns requires a priori setting of model boundary conditions representing the patients global pathology, such as width and morphology of the QRS complex or LV ejection fraction. Even when the global pathology is known and taken into account, large heterogeneities in accuracy and precision exist across the parameter space. For example, late-activated regions will be more difficult to detect when the tissue is also hypocontractile.

### Scalability of proposed parameter space exploration technique

Our findings would be challenging to reproduce using a geometrically detailed finite-element modelling approach due to the large number of simulations required (> 1000 per global pathology). By using the CircAdapt model, with its simplified geometry and consequently faster simulation speed, we were able to perform the 1025 different simulations of cardiac mechanics and hemodynamics per global pathology in < 10 h on a regular PC (64 bit 2.7 GHz CPU and 8 GB RAM). In this study, only two tissue properties were varied that are known to be relevant for the cardiac pathology in patients receiving CRT for heart failure treatment. The number of simulations grows exponentially with the number of studied parameters and could thus become impractical when including additional parameters. Meta-modelling numerical techniques for parameter space exploration can be applied to circumvent this issue. Multivariate regression [35, 36], data-driven reduction [37] and Bayesian sensitivity analysis [38] can all reduce the number of required simulations while preserving parameter space exploration. Efficient sampling schemes such as Latin hypercube sampling and polynomial chaos expansions can also be used to reduce the number of simulations required when exploring model behavior across a high-dimensional parameter space [39, 40].

Another problem which emerges with increased number of studied parameters is difficult to visualize all of the different characteristics of the studied parameter space. While two parameters can be presented in a 2D graph, three present a challenge, any more than three parameters would need to be approached differently, for example using dimensional stacking [41]. Manifold learning could help reduce the dimensionality of the output space of the model, by building a reduced-dimensional basis for it [42]. Statistical learning could then allow the definition of a surrogate model, which would help to infer the properties of the input/output map. In this study, we provided videos as Additional files 1, 2, 3, 4 to visualize parameter uncertainty and the corresponding strain pattern cohorts for each simulated pathology.

### Appropriateness of assumed measurement noise

The similarity of two simulated strain patterns was quantified as the squared difference between the two patterns, averaged over all time points within a predefined systolic range. All strain values in this range were therefore assumed equally important for determining similarity. In the future, a more selective analysis of specific strain characteristics could be considered to improve accuracy and precision for strain-based parameter estimation.

The value of the similarity threshold used in this study (*D*_*i,p*_≤ 0.02) can be considered an absolute strain error of ± 2%. A recent study in subjects with normal cardiac function that compared global longitudinal strain measurements obtained using nine commercial echocardiographic deformation imaging devices [43] reported intra-observer and inter-observer variabilities of global longitudinal strain ranging from 0.9 to 1.7% in absolute strain %points. Furthermore, another study using MRI-tagging to measure LV deformation reported inter-study variability in peak systolic circumferential strain of ± 1.9% in absolute strain %-points [44]. Our 2% similarity threshold thus represents a realistic level of strain measurement error.

An additional potential error that we did not take into account in this study is signal drifting. Typically, the R-top or onset of the QRS complex on the ECG is used as the reference point of zero strain, which approximately corresponds to mitral valve closure. From the reference point onwards the strain measurement can accumulate residual error due to signal drifting that can be up to a relative error of 10% by the time of peak systolic strain [45].

### Model of cardiovascular system dynamics

The simulation results presented in this study should be interpreted as the behavior of a single Hill-type myofiber model (the small element) embedded in a larger model of the heart and circulation. In fact, the mechanical boundary conditions (tension) felt by the myofiber model can be provided by other models of the heart. Therefore, our results are likely to apply to other, more complex, models of the heart and circulation provided they use a similar Hilltype model of myofiber mechanics [13–15]. Various studies have shown that the product of simulations of local myocardial tissue mechanics and global cardiac pump function generated by the used model can be found in measurements of dyssynchronous heart failure [3, 28, 29, 31, 46].

### Clinical implications

Our study reiterates that AT and contractility interact to determine regional myocardial strain. AT may be poorly determined in regions where contractility is low, and regions of relatively high contractility may also be hard to distinguish from one another. Our simulations support the combination of electrical and mechanical measurements in order to reduce the uncertainty in the parameters to be estimated. Furthermore, global mechanical interactions between ventricular walls and between regions within the ventricular walls should be considered when examining regional strain curves.

This study is also a step towards reliable extraction of mechanical and electrical myocardial tissue properties from noninvasive myocardial deformation measurements using a model of cardiac mechanics. Electromechanical simulation-based mapping of local ventricular tissue properties, such as AT and contractility, would enable the clinician to characterise the patients complex heterogeneity of pathological electro-mechanical tissue substrates and to optimise CRT delivery by personalising pacing therapy based upon the individual patients underlying myocardial pathology.

## Conclusion

In this simulation study we demonstrated that similarity of local myocardial strain patterns implies similarity of underlying local activation time and contractility, provided that the global pathological condition is known. These results suggest that we found no theoretical limitation for simulation-based mapping of local myocardial activation time and contractility from myocardial deformation measurements however, heterogeneous distribution of parameter uncertainty throughout the evaluated parameter space should be considered. In contrast, when the global cardiac pathology is not known, similarity of local myocardial strain patterns does not guarantee similarity of underlying local tissue parameters. The relation can become degenerate, with different regions of parameter space producing similar strain patterns. The result is a theoretical constraint on parameter extraction from myocardial deformation due to large uncertainty in the derived parameters.

## Additional files

**Additional file 1.** Video of cohorts obtained when strain patterns are sourced from all four global cardiac pathologies, while parent strain is sourced from HF + HYPO simulation (black dot in parameter space) and runs throughout all 1025 values in the parameter space. The colours of cohort components in the parameter space (left) and strain pattern representation (right) correspond to different global cardiac pathologies: HF (yellow), HF + HYPO (orange/red), HF + LBBB (orange) and HF + LBBB + HYPO (red). Note that cohort members cannot be distinguished in strain pattern representation and that fragmentation of a cohort composition in parameter space signifies system degeneracy.

**Additional file 2.** Video of cohorts obtained when strain patterns are sourced from all four global cardiac pathologies, while parent strain is sourced from HF + LBBB + HYPO simulation (black dot in parameter space) and runs throughout all 1025 values in the parameter space. The colours of cohort components in the parameter space (left) and strain pattern representation (right) correspond to different global cardiac pathologies: HF (yellow), HF + HYPO (orange/red), HF + LBBB (orange) and HF + LBBB + HYPO (red). Note that cohort members cannot be distinguished in strain pattern representation and that fragmentation of a cohort composition in parameter space signifies system degeneracy.

**Additional file 3.** Video of cohorts obtained when strain patterns are sourced from all four global cardiac pathologies, while parent strain is sourced from HF + LBBB simulation (black dot in parameter space) and runs throughout all 1025 values in the parameter space. The colours of cohort components in the parameter space (left) and strain pattern representation (right) correspond to different global cardiac pathologies: HF (yellow), HF + HYPO (orange/red), HF + LBBB (orange) and HF + LBBB + HYPO (red). Note that cohort members cannot be distinguished in strain pattern representation and that fragmentation of a cohort composition in parameter space signifies system degeneracy.

**Additional file 4.** Video of cohorts obtained when strain patterns are sourced from all four global cardiac pathologies, while parent strain is sourced from HF simulation (black dot in parameter space) and runs throughout all 1025 values in the parameter space. The colours of cohort components in the parameter space (left) and strain pattern representation (right) correspond to different global cardiac pathologies: HF (yellow), HF + HYPO (orange/red), HF + LBBB (orange) and HF + LBBB + HYPO (red). Note that cohort members cannot be distinguished in strain pattern representation and that fragmentation of a cohort composition in parameter space signifies system degeneracy.
